# Health outcomes, education, healthcare delivery and quality – 3041. Smoking habit behaviour in adolescents

**DOI:** 10.1186/1939-4551-6-S1-P214

**Published:** 2013-04-23

**Authors:** Belkis López González

**Affiliations:** 1 Allergy Deparment, Clinic: Robert Manuel Zulueta, Cuba

## Background

The main objective of this paper was to identify the percentage of smoking adolescents as well as the tobacco consumption and present asthma symptoms according to sex and age. Smoking habit play an important role in respiratory infections due it is a prenatal conditioning factor for the posterior appearance of asthma in the child and for the worsening of pre-established symptoms in active smokers as well as passive ones. Information related to this health problem is short as well as descriptions regarding asthma in smokers. There are uncountless descriptions about the dangerous effects of smoking cigarettes causing disorders to different organ systems although this paper has focused mainly the respiratory system where tobacco has been involved as the risk factor causing inflammatory infections as well as carcinomas.

## Methods

A cross section study was done using a questionary in which the students answered according to their interpretation. The sample was taken to two hundred and ten participants. The data were collected through a doctor- patient interview and triangulated with information from a questionnaire and the study of individual and family clinical histories. This was organized according to age, sex, tobacco consumption habit, and asthma or related symptoms for the analysis. The students were eighth and ninth graders and were selected according to sex and age.

## Results

Sex distribution showed that 57.61 % were male adolescents and 42.38 % were females of whom 4.76 % presented with smoking habits and none were asthmatic. Sixteen point six, six (16.66) % of their fathers smoked, while the figure for mothers was 12.85 %. The analysis also revealed that 15.23 % of these lived with someone who also smoked. The most commonly asthma-related symptoms found were wheezing, chest tightness and coughing in order of apearence.

## Conclusions

There were no significant differences regarding sex. The occurrence of adolescent smokers is high as well as the population suffering asthma symptoms; therefore, prevention is a key point for the health professionals who are in charge of developing this task in the health care community.

